# The Ameliorating Effects of n-3 Polyunsaturated Fatty Acids on Liver Steatosis Induced by a High-Fat Methionine Choline-Deficient Diet in Mice

**DOI:** 10.3390/ijms242417226

**Published:** 2023-12-07

**Authors:** Václav Šmíd, Karel Dvořák, Kamila Stehnová, Hynek Strnad, Josep Rubert, Jan Stříteský, Barbora Staňková, Milena Stránská, Jana Hajšlová, Radan Brůha, Libor Vítek

**Affiliations:** 1 4th Department of Internal Medicine, 1st Faculty of Medicine, Charles University in Prague and General University Hospital, 128 08 Prague, Czech Republicbruha@cesnet.cz (R.B.); vitek@cesnet.cz (L.V.); 2 Department of Food Analysis and Nutrition, University of Chemistry and Technology, 166 28 Prague, Czech Republic; kamila.stehnova@vscht.cz (K.S.); josep.rubert@wur.nl (J.R.); jana.hajslova@vscht.cz (J.H.); 3 Laboratory of Genomics and Bioinformatics, Institute of Molecular Genetics of the Czech Academy of Sciences, 142 20 Prague, Czech Republic; 4 Institute of Pathology, 1st Faculty of Medicine, Charles University in Prague and General University Hospital, 128 00 Prague, Czech Republic; jan.stritesky@lf1.cuni.cz; 5 Institute of Medical Biochemistry and Laboratory Diagnostics, 1st Faculty of Medicine, Charles University in Prague and General University Hospital, 128 08 Prague, Czech Republic

**Keywords:** non-alcoholic fatty liver disease, non-alcoholic steatohepatitis, n-3 fatty acids, lipidome, lipids

## Abstract

The pathogenesis of non-alcoholic fatty liver disease (NAFLD) is associated with abnormalities of liver lipid metabolism. On the contrary, a diet enriched with n-3 polyunsaturated fatty acids (n-3-PUFAs) has been reported to ameliorate the progression of NAFLD. The aim of our study was to investigate the impact of dietary n-3-PUFA enrichment on the development of NAFLD and liver lipidome. Mice were fed for 6 weeks either a high-fat methionine choline-deficient diet (MCD) or standard chow with or without n-3-PUFAs. Liver histology, serum biochemistry, detailed plasma and liver lipidomic analyses, and genome-wide transcriptome analysis were performed. Mice fed an MCD developed histopathological changes characteristic of NAFLD, and these changes were ameliorated with n-3-PUFAs. Simultaneously, n-3-PUFAs decreased serum triacylglycerol and cholesterol concentrations as well as ALT and AST activities. N-3-PUFAs decreased serum concentrations of saturated and monounsaturated free fatty acids (FAs), while increasing serum concentrations of long-chain PUFAs. Furthermore, in the liver, the MCD significantly increased the hepatic triacylglycerol content, while the administration of n-3-PUFAs eliminated this effect. Administration of n-3-PUFAs led to significant beneficial differences in gene expression within biosynthetic pathways of cholesterol, FAs, and pro-inflammatory cytokines (IL-1 and TNF-α). To conclude, n-3-PUFA supplementation appears to represent a promising nutraceutical approach for the restoration of abnormalities in liver lipid metabolism and the prevention and treatment of NAFLD.

## 1. Introduction

Non-alcoholic fatty liver disease (NAFLD) is one the most common chronic liver diseases in Western countries. The development of NAFLD is associated with lipid dysregulation and abnormalities in the liver, especially changes in the composition of the liver fatty acid (FA) content [1]. The mechanisms responsible for the increase in oxidative stress, inflammation, and hepatocellular damage associated with NAFLD derive from the pathological effects of excessive lipid accumulation [2,3,4,5]. The severity of steatosis correlates well with a risk of progression from NAFLD to non-alcoholic steatohepatitis (NASH) [6]. Exaggerated oxidative stress is an important and probably central mechanism in the progression toward NASH. It leads to a liver inflammatory response, which can eventually result in apoptosis [7]. Saturated free FAs are directly hepatotoxic, mediating an endoplasmic reticulum stress response and inducing hepatocyte apoptosis (called lipoapoptosis or lipotoxicity) [8]. Fat accumulation in the liver is also associated with the depletion of n-3 polyunsaturated FAs (n-3-PUFAs), and patients with NAFLD have a reduced intake of n-3-PUFAs and an increased intake ratio of n-6/n-3-PUFAs [3,9,10]. Disturbances in the ratio of saturated/unsaturated FAs play a causal role in the pathophysiology of NAFLD and subsequent NASH [11].

It has been reported in both animal and human studies that n-3-PUFAs are able to limit triacylglycerol (TAG) deposition in the liver [12,13], ameliorating hepatic steatosis and insulin resistance [14,15]. On the other hand, the European Food Safety Authority (EFSA) discussed the safety of omega-3 long-chain polyunsaturated fatty acids (n-3-LCPUFAs) such as eicosapentaenoic acid (EPA), docosahexaenoic acid (DHA) and docosapentaenoic acid because of some concerns about their adverse effects on human health in case of their excessive intake. Nevertheless, the experts concluded that even in case of supplemental intake of EPA and DHA of up to 5 g a day, they do not increase the risk of reported adverse effects such as bleeding episodes, impaired regulation of glucose levels, or impaired immune [16] function. On the other hand, scientific evidence demonstrates that a diet deficient in n-3-PUFAs with a high n-6/n-3 ratio could induce fatty liver [17,18,19]. In the Western diet, the ratio of n-6/n-3-PUFAs is high, markedly exceeding the target value of 2:1, reaching often >10:1 [20]. Under such conditions, it is not surprising that Western diet is associated with increased risk of NAFLD development [21]. It needs to be emphasized that n-3-PUFAs have anti-inflammatory effects in both rodents [15,22] and humans [23,24], and they also improve dyslipidemia [25]. The beneficial metabolic action of n-3-PUFAs has been demonstrated through modulation of tissue production of eicosanoids and other lipid mediators [26], as well as influencing adiponectin levels [27] and the expression of master transcriptional regulators, such as PPARα [28].

Hence, the aims of our study were to investigate the impact of a diet enriched with n-3-PUFAs on the development of NAFLD using a mouse model of NAFLD and to evaluate possible effects on lipid metabolism and specific gene expressions in correlation with the severity of liver disease.

## 2. Results

### 2.1. The Effect of n-3-PUFAs on NAFLD Development

The administration of MCD resulted in an increase of animal as well as liver weights. Co-administration with n-3-PUFAs abolished the weight gain effect of MCD and resulted in weight comparable to the control group, whereas n-3-PUFA administration itself did not have any effect on either animal or liver weights (Table 1). The food intake did not differ between the groups.

Administration of n-3-PUFAs reduced serum concentrations of cholesterol and TAGs and had specific effects on plasma FA composition. In fact, serum TAG concentrations were significantly decreased upon n-3-PUFA supplementation in both the MCD and control groups (Table 1). The same pattern was found for total serum cholesterol concentrations, which were significantly decreased upon n-3-PUFA treatment, again in both the MCD and control groups (Table 1). Similarly, the total FA concentrations were significantly lower in n-3-PUFA-treated groups, with significantly lower concentrations of saturated FAs and monosaturated FAs (Table 2 and Table 3). The n-6/n-3 ratio of serum FAs was highest in the MCD-fed mice and significantly decreased in the n-3-PUFA-treated animals. As expected, serum concentrations of DHA and EPA correlated with the administration of n-3-PUFAs (Table 3).

**Figure 1:**
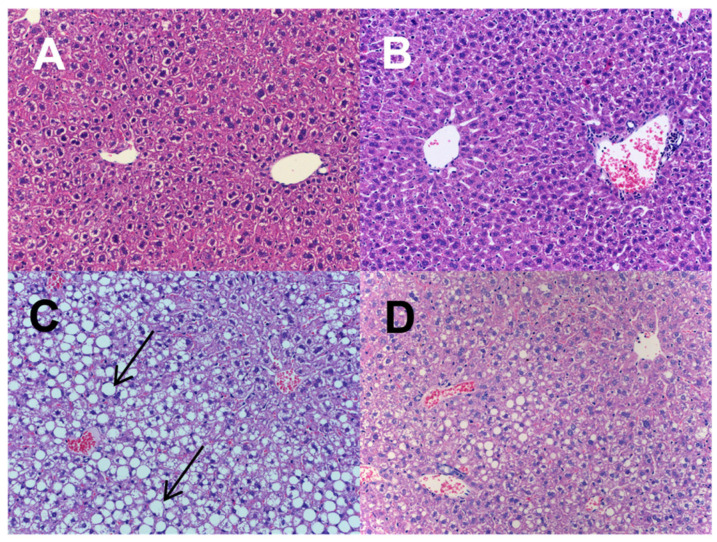
Administration of n-3-PUFAs ameliorated the histopathological signs of NAFLD. Histological assessment of liver was performed after 6 weeks of treatment and the data were statistically analyzed. Representative H and E-stained liver microscopic sections are displayed. The arrows show steatosis characterized by large vacuoles that occupy the whole cytoplasm of hepatocytes, pushing the nucleus to the side of the cell. Dietary interventions regulated hepatic accumulation of lipids. In group M, dominantly zonal periportal macrovesicular steatosis was observed. Panlobular steatosis was found in nine animals from 11 in the M group. The control groups (C, CP) are characterized by the absence of steatosis and ballooning degeneration. (**A**) control; (**B**) control and n-3-PUFAs; (**C**) high-fat methionine choline-deficient diet; (**D**) high-fat methionine choline-deficient diet + n-3-PUFAs.

**Figure 2:**
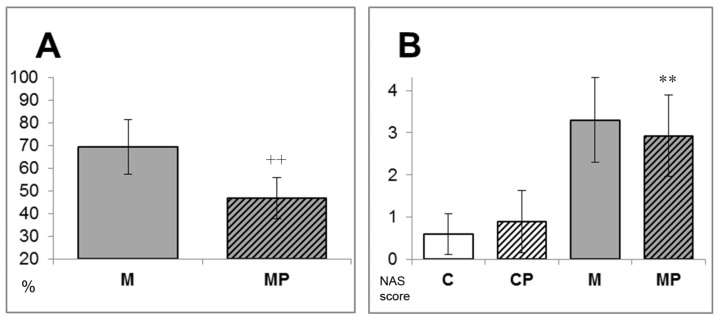
Effect of n-3-PUFA treatment on the degree of steatosis and NAS score in a high-fat MCD NAFLD model. Coadministration with n-3-PUFAs (MP) showed a significant reduction in steatosis and improvement of NAS score as compared to the high-fat methionine choline-deficient-fed mice (M). (**A**) Area of steatosis; (**B**) NAS score, which reflects NAFLD activity, is calculated by summing the scores of steatosis (0–3), lobular inflammation (0–3), and hepatocyte ballooning (0–2) [29]. ** *p* < 0.01 vs. C; ^++^ *p* < 0.01 vs. M; C—control; CP—control and n-3-PUFAs; M—high-fat methionine choline-deficient diet; MP—high-fat methionine choline-deficient diet + n-3-PUFAs.

These observations correlated well with liver lipid content, which was significantly higher in MCD-exposed mice, with amelioration mediated by the n-3-PUFA treatment (Table 2). In addition, MCD administration for 6 weeks resulted in liver injury, as evidenced by a significant elevation in serum ALT activity, while supplementation of n-3-PUFAs completely eliminated this effect (Table 1). The elevation of ALT in mice exposed to MCD was due to histopathological changes consistent with NAFLD development. Indeed, the livers from mice fed an MCD, as well as those of the mice fed an MCD + n-3-PUFA diet, showed both predominantly zonal periportal macrovesicular steatosis and more frequent intralobular hepatocyte apoptosis (Figure 1). However, while panlobular steatosis was found in the majority of the MCD-fed animals (82%), it was found in only 33% of n-3-PUFA-fed mice. The overall degree of steatosis was significantly higher in the MCD group compared to the n-3-PUFA-treated mice (69.4 ± 12% vs. 46.8 ± 9%, *p* < 0.01, Figure 2A). All these histopathological changes were practically absent in both control groups. Intralobular inflammatory infiltrates were observed in both groups fed an MCD, and n-3-PUFAs did not revert these changes.

**Figure 3:**
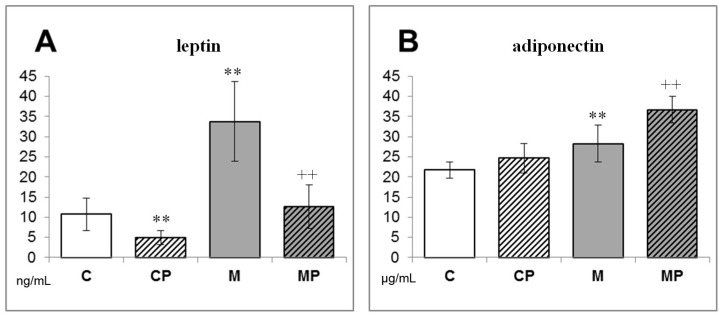
Plasma levels of leptin and adiponectin. Administration of n-3-PUFAs completely normalized plasma leptin levels, which were significantly increased by a high-fat methionine choline-deficient diet. Surprisingly, n-3-PUFAs decreased leptin levels in the control group as well. (**A**) Plasma leptin levels; (**B**) Plasma adiponectin levels. ** *p* < 0.01 vs. C; M; ++ *p* < 0.01 vs. M; C—control; CP—control and n-3-PUFAs; M—high-fat methionine choline-deficient diet; MP—high-fat methionine choline-deficient diet + n-3-PUFAs.

The beneficial effects of n-3-PUFA supplementation were also reflected in the response of plasma concentrations of leptin, which were significantly increased in MCD-fed mice compared to controls (33.8 ± 9.8 vs. 10.7 ± 4.1 ng/L, *p* < 0.001), a phenomenon completely normalized with the co-administration of n-3-PUFAs (33.8 ± 9.8 vs. 12.58 ± 5.4 ng/L, *p* < 0.001). Surprisingly, n-3-PUFAs decreased plasma leptin concentrations even in the control group (10.7 ± 4.1 vs. 4.9 ± 1.8 ng/L, *p* < 0.01, Figure 3A).

### 2.2. The Effect of n-3-PUFAs on Liver Lipidome

Lipidomic analysis of the TAGs, phospholipids, and cholesteryl esters fractions in the liver tissue revealed significant changes in the FA profiles in response to all the interventions.

#### 2.2.1. The Effect of n-3-PUFAs on Liver TAGs

**Figure 4:**
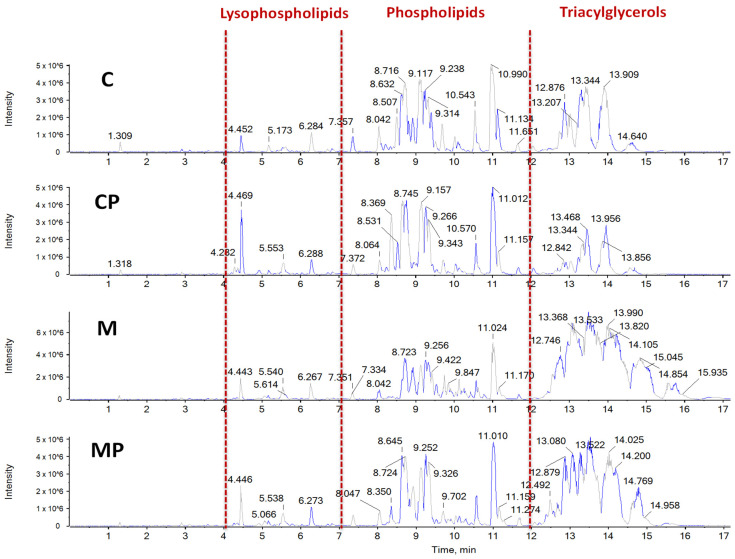
n-3-PUFA significantly decreased liver TAG content. Cyclohexane extracts of mouse livers were measured via positive ionization mode. The most significant differences between samples can be observed at retention times ranging from 12 to 17.5 min, where TAGs were mainly eluted. n-3-PUFA supplementation resulted in a decrease in the intensities of TAGs. After data evaluation, it should be noted that the liver extracts of the mice fed a high-fat MCD (M, MP) contained TAGs with higher m/z values, or in other words, TAGs containing fatty acids with long carbon chains. In addition, when PUFAs were added, the number of double bonds in the fatty acids present in TAGs also increased. C—control; CP—control + n-3-PUFAs; M—MCD; MCD—high-fat methionine choline-deficient diet; MP—MCD + n-3-PUFAs.

**Figure 5:**
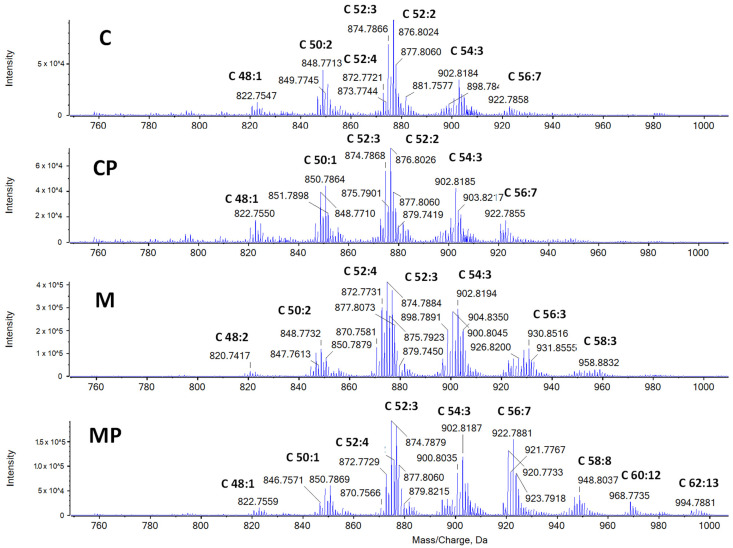
A higher content of TAGs with longer carbon chains was found in the groups with n-3-PUFAs. The mass spectra of TAGs were extracted from the 12 to 17.5 min time window in the chromatogram (Figure 5). After the n-3-PUFA treatment, the number of double bonds in the FAs present in TAGs increased. A higher content of TAGs with longer carbon chains (C58, C60, C62 and C64) was found in the livers of mice fed a high-fat MCD with n-3-PUFAs (group MP) in comparison to group M. Zoomed-out m/z 750-1050 (cyclohexane, positive ionization mode), RT 12-17.5 min. The codes C x:y indicate the total number of carbons (x) and number of double bonds (y) in the fatty acids bond to respective triacylglycerol. C—control; CP—control + n-3-PUFAs; M—MCD; MCD—high-fat methionine choline-deficient diet; MP—MCD + n-3-PUFAs.

MCD significantly increased the TAG content in the liver tissue of both exposed mouse groups. Importantly, n-3-PUFAs in the diet had an ameliorating effect (Figure 4). The livers from the mice fed an MCD contained TAGs with longer carbon chains (Figure 5). A higher content of TAGs with longer carbon chains (C58, C60, C62 and C64) was found in the livers of mice fed an MCD with n-3-PUFAs, as compared to the mice fed only an MCD. The increase was 156% for C58, 288% for C60, and 2362% for C62, with C64 found exclusively in the mice fed n-3-PUFAs. On the contrary, TAGs with shorter carbon chains (C46, C48, and C50) were more abundant in both control groups. In addition, when n-3-PUFAs were added, the number of double bonds in the FAs present in the TAGs increased.

#### 2.2.2. The Effect of n-3-PUFAs on Liver Free FAs

Mainly free FAs were commonly detected in the liver tissue in each mouse group. The essential difference between the diets with and without n-3-PUFAs lies in the main fatty acids present. Hence, while the mice not fed n-3-PUFAs (C, M) had the highest intensity found for arachidonic acid (C 20:4), the mice fed n-3-PUFAs contained high amounts of DHA (C22:6) and EPA (C20:5).

#### 2.2.3. The Effect of n-3-PUFAs on Liver Phospholipids

**Figure 6:**
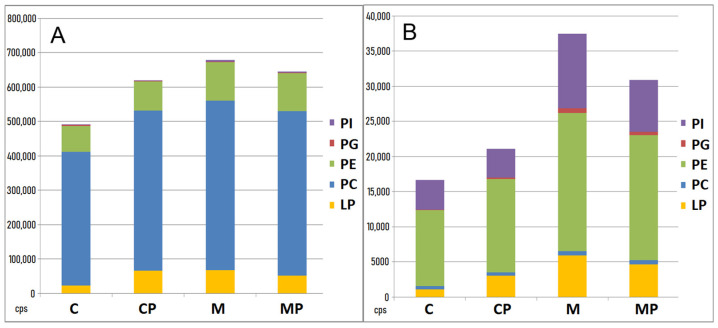
Absolute sum of phospholipids in the liver tissue detected in both positive and negative polarities. Extracts of livers were analyzed using the UHPLC-HRMS technique. (**A**) In the positive ionization mode, the most abundant phospholipids were phosphatidylcholines (PCs) and phosphatidylethanolamines (PEs). n-3-PUFA administration resulted in an increase in the phospholipid content in the chow group (CP vs. C) and a decrease in the MCD group (MP vs. M). (**B**) The most abundant phospholipids found in negative ionization mode (**B**) were phosphatidylethanolamines (PEs) and phosphatidylinositols (PIs); however, phosphatidylglycerols (PGs) and phosphatidylcholines (PCs) were also identified. The same pattern as in the positive ionization mode was observed in negative ionization mode after n-3-PUFA treatment. C—control; CP—control + n-3-PUFAs; cps—counts per second; LP—lysophospholipid; M—MCD; MCD—high-fat methionine choline-deficient diet; MP–MCD + n-3-PUFAs; PE—phosphatidylethanolamine; PI—phosphatidylinositol; PG—phosphatidylglycerol; PC—phosphatidylcholine.

The most abundant phospholipids were phosphatidylcholines and phosphatidylethanolamines, even though phosphatidylglycerols and phosphatidylinositols were also present. The highest content of phospholipids was found in the MCD-fed mice; by contrast, the lowest amounts of liver phospholipids were found in control, untreated animals (Figure 6). Supplementation of n-3-PUFAs decreased the liver phospholipids content in the MCD-fed animals. The most significant differences were found in lysophospholipids. The lowest amount was present in the control, untreated group. In the MCD-fed animals, n-3-PUFAs significantly decreased the amount of liver lysophospholipids (positive ionization mode; total sum 53,096 vs. 70,041 cps, Figure 6A and Supplementary Table S1).

A similar pattern was observed for phospholipids when analyzed in a negative ionization mode. The most abundant phospholipids found in this ionization mode were phosphatidylethanolamines and phosphatidylinositols (Figure 6B). However, phosphatidylglycerols and phosphatidylcholines were also identified. Again, the highest content of these phospholipids was found in the MCD-fed mice; by contrast, the lowest amount was found in the control group (for phosphatidylethanolamines: C 7274, CP 7833, M 13,390, MP 12,830 cps; Supplementary Table S2). Supplementation with n-3-PUFAs led again to a decrease in these phospholipids in the MCD-fed mice, especially phosphatidylinositols (C 4368, CP 4181, M 10,667, MP 7917 cps) and lysophospholipids (C 1175, CP 3248, M 6149, MP 5146 cps; Supplementary Table S2).

### 2.3. Chemometric Analysis PCA-DA of Chromatographic and Spectral Lipidomic Datasets Generated by Metabolic Fingerprinting

The chemometric analysis of lipidomic data revealed TAGs and phospholipids as the most significant markers discriminating individual animal groups according to diet interventions. In fact, the PCA analysis of lipidomics data separated animal groups with dietary interventions into four distinct groups based on the presence of n-3-PUFAs in the diet (with no prior knowledge of sample groups). In the next phase, PCA-DA was employed to determine the variables that maximize the variation between groups and minimize the variation within groups. The analysis was conducted using LipidView and MarkerView tools to assess the influence of individual lipids on the separation among groups.

**Figure 7:**
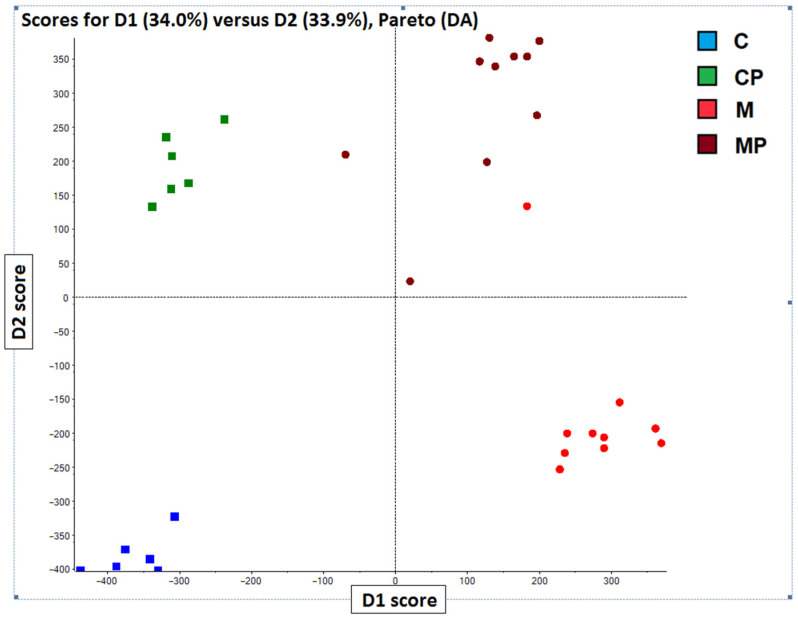
PCA-DA score plot clustering according to the dietary intervention. TAG profiling using PCA-DA analysis. PCA score plot obtained for the cyclohexane extract analyzed by UHPLC-HRMS using negative ionization mode. The samples were clearly clustered into four groups according to the dietary interventions. C—control; CP—control + n-3-PUFAs; M—MCD; MCD—high-fat methionine choline-deficient diet; MP—MCD + n-3-PUFAs.

**Figure 8:**
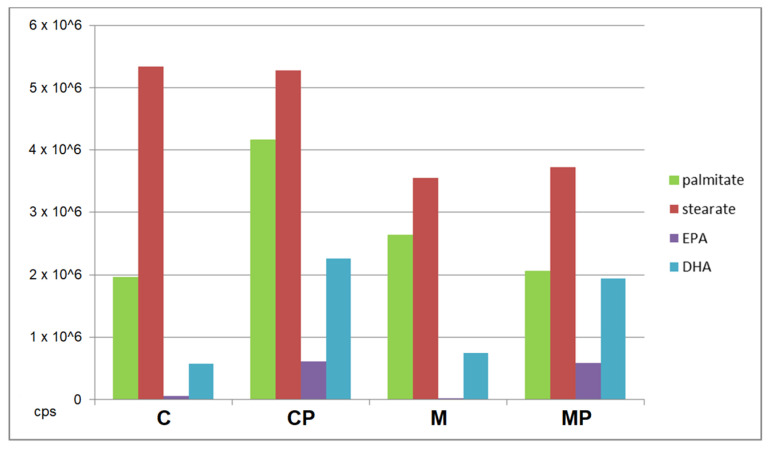
n-3-PUFA supplementation changed the concentrations of cholesteryl esters in the liver. Four cholesteryl esters were identified. The highest intensity (i.e., highest content) of cholesteryl esters was found in the control group with n-3-PUFAs. C—control; CP—control + n-3-PUFAs; DHA—docosahexaenoic acid; EPA—eicosapentaenoic acid; M—MCD; MCD—high-fat methionine choline-deficient diet; MP—MCD + n-3-PUFAs.

The most pronounced differences were observed for TAGs, which substantially improved discrimination of individual groups, especially between mice fed an MCD and those with n-3-PUFA supplementation (Figure 7). In brief, the livers of mice fed MCD showed TAGs with a composition of C 50:4, C 52:4, C 52:5, C 54:2, C 54:5, C 56:3, C 56:4, C 56:5, with fewer carbons and double bonds compared to the MCD mice supplemented with n-3-PUFAs. The supplemented group had more TAGs with C 56:9, C 58:7, C 58:8, and C 58:9. In addition, phospholipids also contributed to the discrimination of the analyzed groups. In contrast to TAGs, phospholipids can be ionized using both positive and negative ionization modes. At this point, both polarities were employed; however, negative ionization mode provided more pronounced clustering and additional information for phospholipid profiling. Furthermore, significant changes were also found in the concentrations of cholesteryl esters in the livers. In fact, four cholesteryl esters were significantly different, including cholesteryl stearate, cholesteryl palmitate, as well as cholesteryl EPA and cholesteryl DHA, with the latter two being the most represented in the n-3-PUFA-supplemented mice (Figure 8).

### 2.4. The Effect of n-3-PUFAs on Expression of Specific Genes in the Liver Tissue

**Figure 9:**
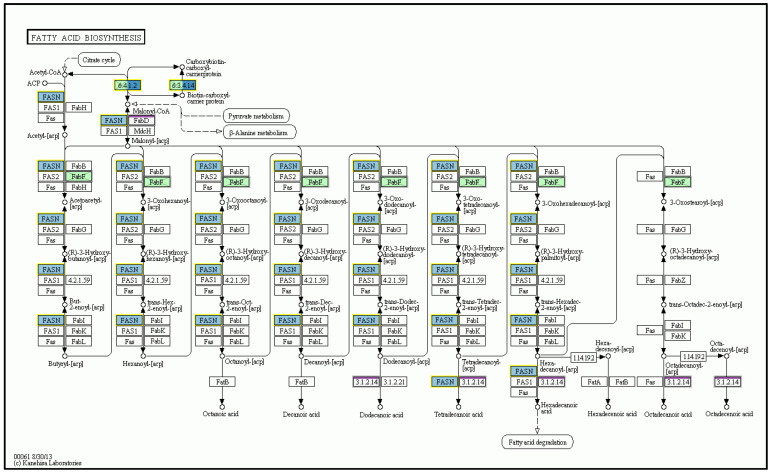
N-3-PUFAs decreased mRNA expression of key enzymes of fatty acid biosynthetic pathway. KEGG pathway map of fatty acid biosynthesis pathway including acetyl-CoA carboxytransferase [EC 2.1.3.15] and FA synthase [EC:2.3.1.85]. MP vs. M. M—MCD; MCD—high-fat methionine choline-deficient diet; MP—MCD + n-3-PUFAs. © Kanehisa Laboratories.

**Figure 10:**
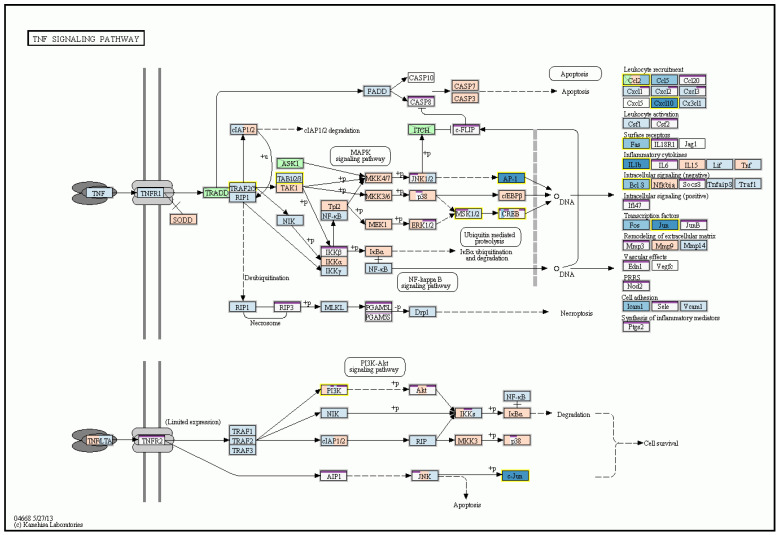
n-3-PUFA administration decreased mRNA expression of pro-inflammatory cytokines, as documented in the TNF signaling pathway. KEGG pathway map of the TNF-α signaling pathway. n-3-PUFA supplementation decreased mRNA expression of pro-inflammatory cytokines (IL-1β), as well as genes responsible for leukocyte recruitment, surface receptors, intercellular signaling (Bcl3), and transcription factors (Jun); MP vs. M. M—MCD; MCD—high-fat methionine choline-deficient diet; MP—MCD + n-3-PUFAs. © Kanehisa Laboratories.

**Figure 11:**
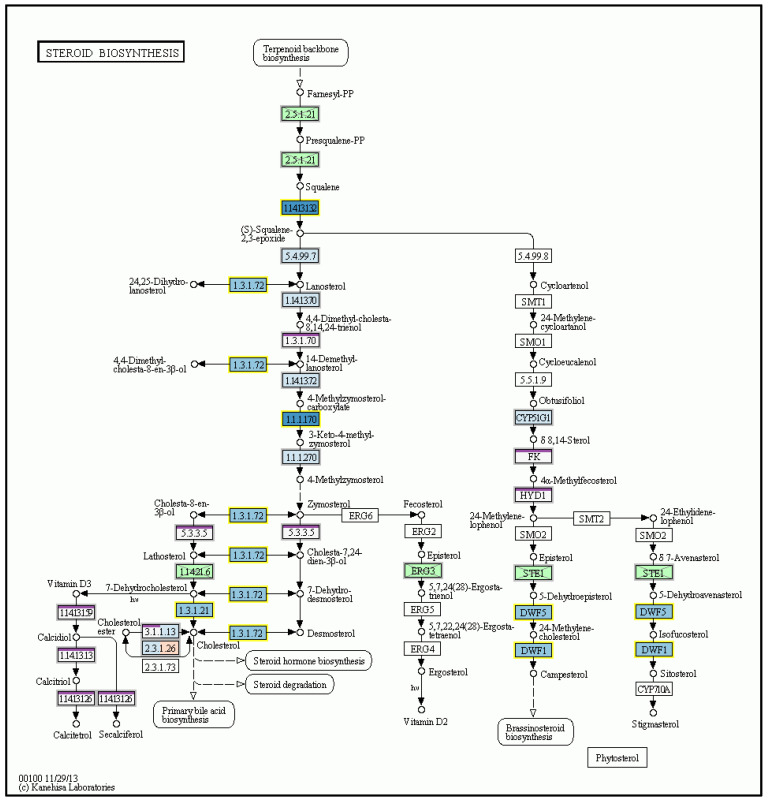
Supplementation of n-3-PUFAs also led to a significant decrease in the mRNA expression of genes of the steroid biosynthetic pathway. KEGG pathway map of steroid biosynthesis. mRNA expression of squalene monooxygenase [EC:1.14.14.17], sterol-4α-carboxylate 3-dehydrogenase [EC:1.1.1.170], delta24-sterol reductase [EC:1.3.1.72], and 7-dehydrocholesterol reductase [EC:1.3.1.21] was significantly decreased by n-3-PUFA administration; MP vs. M. M—MCD; MP—MCD + n-3-PUFA. © Kanehisa Laboratories.

Detailed microarray analysis revealed differences in liver mRNA transcriptomes after MCD as well as n-3-PUFA administration. As much as 126 gene transcripts were significantly changed (at least a twofold change, *q* < 0.05) between MCD-fed mice and the same animals supplemented with n-3-PUFAs. Importantly, the administration of n-3-PUFAs influenced the expression of genes coding for enzymes of FA biosynthesis pathways, including a significant decrease in mRNA expression of acetyl-CoA carboxytransferase [EC 2.1.3.15], FA synthase [EC:2.3.1.85], and other genes involved in the FA biosynthesis, FA elongation (Figure 9), as well as FA degradation. Hepatic gene expression encoding key cytokines were also significantly changed, with pro-inflammatory cytokines IL-1 and TNF-α being significantly decreased after n-3-PUFA administration (Figure 10). Supplementation of n-3-PUFAs also led to a significant decrease in the mRNA expression of genes of the steroid biosynthetic pathway (e.g., squalene monooxygenase [EC:1.14.14.17], sterol-4α-carboxylate 3-dehydrogenase [EC:1.1.1.170], delta24-sterol reductase [EC:1.3.1.72], and 7-dehydrocholesterol reductase [EC:1.3.1.21]) (Figure 11).

## 3. Discussion

NAFLD is a complex disorder with several subtypes [30] differing in deterioration/impairment of lipid metabolism pathways [31]. Derangement of the metabolism of TAGs, phospholipids, and other lipids is well known in murine models as well as human studies [32,33]. A Western diet, rich in saturated FAs and processed sugars but deficient in n-3-PUFAs, along with a high n-6/n-3 ratio, is associated with the development of NAFLD and further disease progression [34,35,36]. There is increasing evidence that disorders of lipid metabolism are related to the progression of NAFLD [37]. Particularly high levels of plasma FAs and the subsequent excessive influx of FAs into hepatocytes lead to an overload of the capacity of hepatocytes to manage and export FAs as TAGs. These processes result in increased ER and oxidative stress, lipoapoptosis, autophagy, and inflammation [38,39]. As mentioned before, it has been proven that n-3-PUFAs are depleted in the liver tissue of patients with NAFLD [2,19]. Compared to n-6, n-3-PUFAs show important antiproliferative, anti-inflammatory and modulatory effects on the metabolic and immune systems [40]. Low levels of n-3-PUFAs disturb the balance of circulating lipid levels and may promote steatosis, inflammation, dyslipidemia, and an increase in carcinogenesis risk [41,42].

The data from our study on mice fed MCD demonstrate dynamic changes of the plasma and liver lipidome in response to n-3-PUFA supplementation. Quite surprisingly, apparent changes in basic anthropometric and laboratory parameters were also observed, as the body and liver weight were positively affected by n-3-PUFA treatment. Furthermore, we recorded a beneficial biochemical response in ALT activity, an important marker for assessing the severity of NAFLD/NASH and subsequent metabolic complications [43]. A high-fat diet, as well as the MCD used in our study, is associated with the onset of lipotoxicity-induced oxidative stress with progressively increasing availability and oxidation of FAs in the liver [44,45] and increased mitochondrial ROS production induced by increased TNF-α [46]. These pathologic conditions are closely related to an increased n-6/n-3 ratio and depletion of n-3-PUFAs in the liver, leading to insulin resistance [47] and de novo synthesis of saturated FAs [48].

In our study, administration of n-3-PUFAs led to a significant reduction in plasma concentrations of cholesterol and TAGs, associated with a significant reduction in hepatic steatosis, as evidenced by improvement in the histopathology picture as well as total liver fat content. This beneficial effect of n-3-PUFAs on adiposity can be due to n-3-PUFA-mediated PPAR-α activation favoring FA oxidation [49]. In addition, n-3-PUFAs reduced plasma concentrations of total as well as saturated and monosaturated FAs. The plasma n-6/n-3 ratio significantly decreased in n-3-PUFA-treated animals and moved toward control values. It has been demonstrated that an optimal n-6/n-3 ratio suppresses the expression of lipid metabolism-related genes and proteins (such as FA transport protein-1, phosphoinositide-3-kinase-α, and PPAR-γ). In addition, it reduces the expression levels of pro-inflammatory cytokines (TNF-α, IL-1β, and IL-6) [50,51]. Our findings correlate with these observations. The hypolipidemic effects of n-3-PUFAs, together with increased FA oxidation and suppression of hepatic lipogenesis, seem to be associated with versatile metabolic and gene expression changes [14,52].

We have proven that n-3-PUFA administration prevented accumulation of fat in the liver. The beneficial effect of an n-3-PUFA-enriched diet appears to be due to a decrease in saturated, monosaturated, and n-6 FAs in the plasma, substantially preventing the accumulation of fat in the liver. On the other hand, enrichment of n-3-PUFAs (EPA and DHA) exerts their potentially anti-inflammatory and anti-fibrotic effects, as documented in experimental studies [53,54]. Our observations support these data reported previously, as exemplified by their impact on the FA composition of plasma, as well as liver lipidome, and leptin levels.

It has been demonstrated that the combination of metabolic fingerprinting with appropriate chemometric analysis is a valuable approach for differentiating various stages of NAFLD [55,56,57]. These complex lipidomic methods can provide additional insight into the mechanisms of NAFLD development. A unique UHPLC-HRMS/MS lipidomic approach allowed us to investigate detailed changes in the composition of the liver and plasma lipidome. Detailed lipidomic analysis using PCA-DA revealed that n-3-PUFA administration significantly affected the liver lipidomic profile across all lipid subgroups of mice with NAFLD. This analysis separated animal groups into four completely distinct groups based on the presence of n-3-PUFAs in the diet, with TAGs and phospholipids being the most discriminating factors.

In addition to the prevention of liver steatosis induced by MCD, n-3-PUFA supplementation reduced hepatic total fat and TAG content as well as FA and TAG composition. Dietary n-3-PUFA supplementation increased the content of FAs (mostly in TAGs) with longer carbon chains, with C64 found exclusively in this group, enriched by DHA and EPA, respectively. This enrichment may increase/enhance the anti-inflammatory effect of n-3-PUFAs, mediated by resolvins and protectins—metabolites derived from DHA and EPA [58].

n-3-PUFA administration also resulted in significant changes in the profiles of phospholipids and cholesteryl esters fractions. n-3-PUFA-enriched phospholipids (phosphatidylcholine, phosphatidylethanolamine, and lysophosphatidylcholine) with EPA and DHA may lead to decreased lipogenesis, as demonstrated by Lamaziere et al. in a study on rats fed n-3-PUFAs [59] and by Spahis et al. in a human study with NAFLD patients treated with n-3-PUFAs [60]. Our observations align with these previously reported findings, as documented by the results of transcriptome data analysis. The most enhanced differences were observed in lysophospholipids, where n-3-PUFAs completely abolished the negative effect of MCD. Lysophospholipids act as signaling molecules modulating important processes such as inflammation, uptake of glucose (by cultured adipocytes) [61], pancreatic insulin release [62], and insulin sensitivity through their interaction with G-protein-coupled receptors [63]. The suppression of these metabolites by n-3-PUFAs may prevent obesity-related insulin resistance and strongly support the potential therapeutical use of n-3-PUFAs in the treatment of NAFLD.

There are several limitations of our study. Firstly, six weeks of MCD intervention did not result in the development of significant ballooning in the liver parenchyma (typical for NASH), most likely due to the relatively short duration of the intervention. Secondly, we did not analyze the effects of various ratios of EPA and DHA in murine models, which seem to be important. In fact, recent studies indicate that an increased EPA:DHA ratio is favorable for the reduction of pro-inflammatory markers [64,65,66] and accompanied liver injury [67]. Our study was performed with a similar EPA:DHA ratio of 1.3:1, but the optimal composition needs to be confirmed in further studies.

## 4. Materials and Methods

### 4.1. Chemicals

Paraformaldehyde, hematoxylin-eosin, RNA-later, MgSO_4_ (purity ≥ 99%), and all the HPLC-grade solutions and mobile phase modifiers were purchased from Sigma-Aldrich (St. Louis, MO, USA). Deionized water was obtained from a Milli-Q^®^ Integral system supplied by Merck (Darmstadt, Germany). HPLC-grade cyclohexane, acetonitrile, formic acid, and ammonium formate (purity ≥ 99%), as well as NaCl (purity ≥ 99%), were supplied by Penta (Prague, Czech Republic). All other chemicals were purchased locally from Penta (Czech Republic).

### 4.2. Animals and Experimental Design

Male C57BL/6 mice (30 g, aged 3 months), obtained from Anlab (Prague, Czech Republic), were housed under controlled temperature and a natural 12:12 light-dark cycle. All protocols were approved by the Animal Research Committee of the 1st Faculty of Medicine, Charles University, Prague, Czech Republic, and all aspects of the study met the accepted criteria of experimental use of laboratory animals.

### 4.3. Induction of Liver Steatosis

Adult male mice were divided into 4 groups. Two groups of animals representing a NAFLD model were fed a high-fat methionine choline-deficient (MCD) diet (E15652-94 EF R/M, high-fat MCD; 32 kJ% fat, 56 kJ% carbohydrates, 12 kJ% protein, ssniff, Soest, Germany) for 6 weeks (n = 10) [68], each group receiving either n-3-PUFAs (MP) or saline (M) for the whole experiment period. NAFLD control groups (n = 6) were fed chow containing n-3-PUFAs (CP) or saline (C) in the same dose as the MCD-fed animals.

### 4.4. N-3-PUFA Administration

N-3-PUFAs were obtained from Farmax (Hradec Králové, Czech Republic). Each animal received a daily dose of either 0.1 mL of a mixture containing docosahexaenoic acid (DHA; 22:6n-3; 2.4 mg/kg) and eicosapentaenoic acid (EPA; 20:5n-3; 3.2 mg/kg) (56.25 mg EPA + 42.5 mg DHA) via an orogastric tube or saline.

### 4.5. General Tissue Preparation

The mice were anesthetized through intramuscular application of ketamine (100 mg/kg) and xylazine (16 mg/kg). The peritoneal cavity was opened, and blood was taken from the inferior vena cava. The liver was removed, weighted, and cut into samples (paraformaldehyde, liquid nitrogen, and RNA later).

### 4.6. Serum Biochemistry

Serum biochemical markers (total bilirubin, alanine aminotransferase (ALT), aspartate aminotransferase (AST), alkaline phosphatase (ALP), total cholesterol, and TAGs were determined through standard assays using an automatic analyzer (Modular analyzer, Roche Diagnostics, Mannheim, Germany).

### 4.7. Liver Histology

Two small liver tissue blocks (about 1 cm^3^) were fixed in 4% paraformaldehyde, followed by a standard procedure for paraffin embedding. Serial sections, 5–7 µm thick, were cut and stained with hematoxylin and eosin. The slides were viewed using standard light microscopy. Histological assessment was performed by an experienced liver histopathologist according to Kleiner [29]. From a representative section of each tissue block, its area was determined, and the NAS score was established by Kleiner (steatosis, ballooning, and intralobular inflammation). The ratio of micro/macrovesicular steatosis was obtained. The following markers were searched for: dissolution of hepatocytes, portal and periportal inflammatory infiltrates, presence of Mallory-Denk bodies, and signs of fibrosis.

### 4.8. Determination of Adipokines

The concentrations of adiponectin and leptin in mice sera were measured using sandwich enzyme immunoassays, with the spectrophotometric detection at 450 nm (ELISA kits, Uscn Life Science Inc., Wuhan, China).

### 4.9. Serum FA Composition

Total serum FAs were extracted from the serum (50 mL), as described previously [69], using dichloromethane instead of chloroform [70] and then analyzed by GC using a Trace-GC gas chromatograph with a flame ionization detector (Thermo-Finnigan, San Jose, CA, USA). Analyses of fatty-esther methyl esthers (FAME) were performed on a fused-silica capillary column coated with a chemically bond stationary phase CP-Sil 88 CB (Agilent Technologies, Santa Clara, CA, USA). Integration software Clarity version 2.4.1.57 (Prague, DataApex, Czech Republic) was used for data acquisition and handling.

### 4.10. Lipidomic Analysis

Ultra-high-performance liquid chromatography coupled to high-resolution tandem mass spectrometry (UHPLC-HRMS/MS) was used for analyses of mouse liver tissue samples. The instrumentation platform employed for analyses of both acetonitrile and cyclohexane extracts consisted of the Dionex UltiMate 3000 UHPLC system (Thermo Fisher Scientific, Waltham, MA, USA) coupled to the quadrupole-time-of-flight AB SCIEX TripleTOF^®^ 5600 mass spectrometer (AB SCIEX, Vaughan, ON, Canada), as described previously [71], with the following modification of the elution gradient used in both ESI polarities: 0.0 min (90% A; 0.4 mL min^−1^), 1.0 min (50% A; 0.4 mL min^−1^), 5.0 min (80% A; 0.4 mL min^−1^), 11.0 min (0% A; 0.5 mL min^−1^), 19.5 min (0% A; 0.5 mL min^−1^), 20 min (90% A; 0.4 mL min^−1^), 5 min equilibration time (90% A; 0.5 mL min^−1^). Molecular formula estimation, structural elucidation, and subsequent tentative identification of compounds were determined based on MS and MS/MS accurate mass spectra using PeakView 2.0 software (AB Sciex, Concord, ON, Canada) with FormulaFinder.

### 4.11. Transcription Profiling of the Liver Tissues

Total RNA was isolated using the RNeasy Micro Kit (Qiagen, Germantown, MD, USA) from the RNAlater (Ambion, Austin, TX, USA) preserved samples. RNA integrity was analyzed via Agilent Bioanalyzer 2100 (Agilent, Santa Clara, CA, USA). Illumina MouseRef-8 v2.0 Expression BeadChips (Illumina, San Diego, CA, USA) were used for the microarray analysis following the standard protocol. In brief, RNA was amplified with the Illumina TotalPrep RNA Amplification Kit (Ambion, CA, USA) and 1500 ng of labeled RNA was hybridized on the chip according to the manufacturer’s procedure. The analysis was performed with six biological replicates per group. The raw data were preprocessed using GenomeStudio software (version 1.9.0.24624; Illumina, San Diego, CA, USA) and analyzed within the limma package [72] of the Bioconductor [73]: the transcription profiles were background-corrected using a normal-exponential model, quantile normalized, and variance stabilized using a base 2 logarithmic transformation. Moderated *t*-test was used to detect differentially expressed transcripts requiring Storey’s *q* < 0.05 and at least a twofold change in expression. To identify significantly perturbed pathways, we performed a gene set enrichment analysis on KEGG pathways [74].

### 4.12. Statistical Analysis and Data Processing

Normally distributed data are presented as mean ± SD and analyzed using the Student *t*-test. The Mann–Whitney U test or Kruskal–Wallis test was used for non-normally distributed data. Differences with *p* < 0.05 were considered significant. The statistical analyses were performed using Statistica CZ v.12 (StatSoft, Tulsa, OK, USA), unless otherwise stated.

The plasma lipidomic data were processed with the LipidMatch suite, which uses MZmine 2 for feature extraction and an R script for lipid identification based on MS/MS in silico libraries. Statistical analysis of lipidomics data was performed in both web-based and R-based MetaboAnalyst. Before building the statistical models, sum normalization, logarithmic transformation, and Pareto scaling were applied to ensure normal distribution and higher significance of low-abundant compounds. First, the data were evaluated using Principal Component Analysis-Discriminant Analysis (PCA-DA). In principle, large chromatographic and spectral datasets generated by metabolic fingerprinting were used to assess the differences between the groups. Initially, the cyclohexane and acetonitrile extracts analyzed by UHPLC-HRMS/MS were evaluated in both positive and negative polarities. While neutral TAGs were expected to be contained mainly in the nonpolar solvent represented by cyclohexane, more polar lipids such as phospholipids were assumed to be transferred mainly into acetonitrile. Afterwards, the most relevant metabolites, specifically lipid species, were independently evaluated. Based on this strategy, metabolic fingerprinting and profiling provided discrimination power and information in order to understand the influence of fat content and its composition in mouse liver. LipidView (version 1.3 beta) and MarkerView (version 1.2.1) (AB SCIEX, Concord, ON, Canada) tools were used to assess lipid influence and separation among groups. LipidView was individually performed for TAGs and phospholipids (PE—phosphatidylethanolamine; PC—phosphatidylcholine; PI—phosphatidylinositol; PG—phosphatidylglycerol; LP—lysophospholipid). Different filters and databases were used for data processing. Afterwards, the results obtained were exported to MarkerView and statistically processed. As the next step, tools of univariate and multivariate statistics were applied to find and describe variables important for changes in the lipidome.

*T*-test was used to filter out all insignificant features (FDR *p*-value < 0.01) in order to reduce the feature-to-sample ratio before multivariate analysis. A selected subset of lipids was loaded into SIMCA software (v. 13.0, 2011, Umetrics, Umea, Sweden) to build an orthogonal partial least squares discriminant analysis (OPLS-DA) model. A Variable Importance Plot (VIP) score higher than 1 was required to include the feature in the final markers list. The value of the area under the curve (AUC) from the Receiver Operating Characteristic Curve (ROC) was calculated for each variable on the final list to assess its classification strength.

## 5. Conclusions

We conclude that n-3-PUFAs may play an important role in the pathophysiology of NAFLD. In summary, n-3-PUFAs have favorable effects on histopathological changes and serum markers of liver damage. In addition, we have demonstrated dynamic changes of the plasma and liver lipidome after n-3-PUFA supplementation. n-3-PUFA administration changed the metabolomic profile of liver tissue and had a beneficial impact on hepatic lipid content. We expect that n-3-PUFAs may represent a promising nutraceutical approach for restoration of abnormalities of liver lipid metabolism and the prevention and treatment of NAFLD, which should be further studied in clinical trials.

## Figures and Tables

**Table 1:** The effect of n-3-PUFA supplementation on selected serum chemistry parameters.

	C	CP	M	MP
body weight [g]	31.6 ± 1.2	30.7 ± 2.4	34.9 ± 2.6 **	31.2 ± 1.2 ^++^
liver weight [g]	1.38 ± 0.14	1.45 ± 0.24	1.75 ± 0.14 **	1.39 ± 0.13 ^++^
bilirubin [μmol/L]	1.85 ± 0.65	1.30 ± 0.28	1.72 ± 0.5	1.79 ± 0.65
ALT [μkat/L]	0.64 ± 0.2	0.52 ± 0.12	1.29 ± 0.35 **	0.83 ± 0.15 ^+^
AST [μkat/L]	1.65 ± 0.37	1.08 ± 0.19 **	1.43 ± 0.33	1.38 ± 0.5
ALP [μkat/L]	0.73 ± 0.07	0.78 ± 0.13	0.95 ± 0.15 **	0.83 ± 0.15
cholesterol [mmol/L]	2.02 ± 0.12	1.49 ± 0.15 **	3.28 ± 0.62 **	2.36 ± 0.29 ^++^
TAG [mmol/L]	1.06 ± 0.06	0.86 ± 0.16 *	0.85 ± 0.23	0.59 ± 0.16 ^+^
total protein [g/L]	44.6 ± 2.0	44.1 ± 1.9	46.5 ± 3.2	46.1 ± 2.5

Administration of a high-fat MCD led to a significant increase in total body weight. Supplementation of n-3-PUFAs decreased total body weight as well as liver weight after 6 weeks of experiment. Significant decreases in ALT activity, cholesterol and TAG concentrations were observed after n-3-PUFA administration in NAFLD groups (MP vs. M). Data are expressed as mean ± SD. * *p* < 0.05 vs. C; ** *p* < 0.01 vs. C; ^+^ *p* < 0.05 vs. M; ^++^ *p* < 0.01 vs. M; C—control, CP—control and n-3-PUFAs; M—high-fat MCD; MCD—methionine choline-deficient diet; MP—MCD and n-3-PUFAs; TAG—triacylglycerols.

**Table 2:** The effect of n-3-PUFA supplementation on plasma fatty acid composition and total liver lipid content.

	C	CP	M	MP
sum of FAs [μmol/mL]	7.26 ± 1.0	5.97 ± 0.4 *	9.08 ± 1.4 *	6.56 ± 1.4 ^++^
saturated FAs [μmol/mL]	2.36 ± 0.3	1.91 ± 0.1 **	2.47 ± 0.4	1.8 ± 0.3 ^++^
monosaturated [μmol/mL]	1.76 ± 0.3	1.51 ± 0.1	1.82 ± 0.3	1.07 ± 0.4 ^++^
sum of n-6 [μmol/mL]	2.63 ± 0.4	1.88 ± 0.1 **	4.13 ± 0.7 **	2.69 ± 0.6 ^++^
sum of n-3 [μmol/mL]	0.48 ± 0.1	0.65 ± 0.1 *	0.28 ± 0.1 **	0.72 ± 0.1 ^++^
n-6/n-3 ratio	5.5	2.9	14.7	3.7
total liver lipid [g]	3.08 ± 0.36	3.08 ± 0.56	13.97 ± 3.36 **	8.29 ± 3.57 ^++^

The total FA concentrations were significantly lower in n-3-PUFA-treated groups, with significantly lower concentrations of saturated and monosaturated FAs. N-3-PUFAs were significantly decreased in MCD-fed mice compared to controls, a phenomenon completely normalized with co-administration of n-3-PUFAs. The n-6/n-3 ratio of serum FAs was the highest in the MCD-fed mice and significantly decreased in n-3-PUFA-treated animals. Data are expressed as mean ± SD. * *p* < 0.05 vs. C; ** *p* < 0.01 vs. C; ^++^ *p* < 0.01 vs. M; C—control, CP—control and n-3-PUFAs, DHA—docosahexaenoic acid, EPA—eicosapentaenoic acid, FAs—fatty acids, M—high-fat methionine choline-deficient diet (MCD); MP—MCD and n-3-PUFAs.

**Table 3:** Detailed analysis of serum FA concentrations.

[μmol/mL]	C	CP	M	MP
12:0	0.0062 ± 0.003	0.0075 ± 0.003	0.0062 ± 0.001	0.0067 ± 0.003
14:0	0.0283 ± 0.005	0.0273 ± 0.004	0.0205 ± 0.002 **	0.0216 ± 0.009
14:1n-5	0.0016 ± 0.001	0.0017 ± 0.001	0.0008 ± 0.0006	0.0011 ± 0.0005
16:0	1.681 ± 0.23	1.395 ± 0.09 *	1.514 ± 0.23	1.089 ± 0.21 ^++^
16:1n-9	0.043 ± 0.006	0.036 ± 0.003 *	0.029 ± 0.005 **	0.033 ± 0.032
16:1n-7	0.272 ± 0.048	0.243 ± 0.022	0.116 ± 0.029 **	0.074 ± 0.028 ^+^
18:0	0.625 ± 0.05	0.464 ± 0.03 **	0.911 ± 0.14 **	0.664 ± 0.1 ^++^
18:1trans	0.022 ± 0.006	0.016 ± 0.005	0.374 ± 0.13 **	0.290 ± 0.12
18:1n-9c	1.237 ± 0.22	1.052 ± 0.11	1.49 ± 0.21	0.869 ± 0.29 ^++^
18:1n-7c	0.172 ± 0.03	0.147 ± 0.01	0.165 ± 0.03	0.08 ± 0.03 ^++^
18:2n6cc	1.793 ± 0.26	1.474 ± 0.14 *	3.0 ± 0.47 **	2.29 ± 0.59 ^+^
18:3n-6alc	0.025 ± 0.004	0.014 ± 0.003 **	0.015 ± 0.002 **	0.009 ± 0.001 ^++^
18:3n-3alc	0.039 ± 0.008	0.0317 ± 0.007	0.011 ± 0.002 **	0.008 ± 0.002 ^+^
20:0	0.023 ± 0.003	0.016 ± 0.003 *	0.021 ± 0.004	0.016 ± 0.003 ^+^
20:1n-9c	0.0375 ± 0.01	0.032 ± 0.007	0.021 ± 0.004 **	0.014 ± 0.005 ^+^
20:2n-6cc	0.027 ± 0.005	0.023 ± 0.002	0.043 ± 0.008 **	0.012 ± 0.003 ^++^
20:3n-6alc	0.095 ± 0.016	0.076 ± 0.01 *	0.169 ± 0.037 **	0.056 ± 0.011 ^++^
20:4n-6alc	0.668 ± 0.115	0.288 ± 0.046 **	0.869 ± 0.233	0.313 ± 0.131 ^++^
20:5n-3alc	0.043 ± 0.008	0.117 ± 0.037 **	0.016 ± 0.006 **	0.195 ± 0.063 ^++^
22:4n-6alc	0.011 ± 0.002	0.004 ± 0.001 **	0.012 ± 0.002	0.002 ± 0.002 ^++^
22:5n-6	0.012 ± 0.003	0.003 ± 0.001 **	0.022 ± 0.005 **	0.004 ± 0.002 ^++^
22:5n-3alc	0.027 ± 0.004	0.05 ± 0.01 **	0.012 ± 0.007 **	0.038 ± 0.006 ^++^
22:6n-3alc	0.37 ± 0.07	0.452 ± 0.05 *	0.243 ± 0.09 **	0.478 ± 0.07 ^++^

N-3-PUFAs had beneficiary effects on plasma fatty acid composition. As expected, serum concentrations of DHA and EPA correlated with administration of n-3-PUFAs. Data are expressed as mean ± SD. * *p* < 0.05 vs. C; ** *p* < 0.01 vs. C; ^+^ *p* < 0.05 vs. M; ^++^ *p* < 0.01 vs. M; C—control, CP—control and n-3-PUFAs, DHA—docosahexaenoic acid; EPA—eicosapentaenoic acid; M—high-fat methionine choline-deficient diet. MP—MCD and n-3-PUFAs.

## Data Availability

The raw data used to support the findings of this study are available from the corresponding author upon request. The transcriptomic data are available in the ArrayExpress database under the accession E-MTAB-13399 (https://www.ebi.ac.uk/biostudies/arrayexpress/studies/E-MTAB-13399, accessed on 8 October 2023).

## Supplementary Materials

The following supporting information can be downloaded at: https://www.mdpi.com/article/10.3390/ijms242417226/s1.

## Abbreviations

ALT—alanine aminotransferase; AST—aspartate aminotransferase; EC—esters of cholesterol; EPA—eicosapentaenoic acid; DHA—docosahexaenoic acid; FAs—fatty acids; FW—fresh weight; IL—interleukin; MCD—high-fat methionine choline-deficient diet; n-3-PUFAs—omega-3 polyunsaturated fatty acids; n-3-LCPUFAs—omega-3 long-chain polyunsaturated fatty acids; NAFLD—non-alcoholic fatty liver disease; NASH—non-alcoholic steatohepatitis; PUFAs—polyunsaturated fatty acids; TAGs—triacylglycerols; TNF—tumor necrosis factor; UHPLC-HRMS/MS—ultra-high performance liquid chromatography coupled with high resolution tandem mass spectrometry.
