# Supplementary material for: The Ameliorating Effects of n-3 Polyunsaturated Fatty Acids on Liver Steatosis Induced by a High-Fat Methionine Choline-Deficient Diet in Mice
Source: Int J Mol Sci. 2023 Dec 7;24(24):17226. doi: 10.3390/ijms242417226 (PMC10743075; doi:10.3390/ijms242417226)
Supplement: Supplementary file 1 [file ijms-24-17226-s001.zip › ijms-2719308-supplementary.pdf]

Supplementary Materials

Table S1. The effect of n-3-PUFA on liver phospholipids - positive mass spectrometry ionzation mode.

	C		CP		M		MP	
	average intensity	SD (%)	average intensity	SD (%)	average intensity	SD (%)	average intensity	SD (%)
**LPC 16:0**	5686.2	29.0	23702.3	38.0	12517.9	34.0	11849.5	39.4
**LPC 16:1**	0.0		1129.9	71.6	969.5	81.2	584.6	41.0
**LPC 18:0**	2434.3	20.9	8006.9	48.1	10420.4	50.1	10991.2	45.2
**LPC 18:1**	1144.6	23.9	4539.4	42.7	9781.3	61.3	4852.9	46.9
**LPC 18:2**	1004.1	9.1	5300.5	31.8	4102.4	80.5	1981.6	43.2
**LPC 18:3**	1285.5	20.6	3088.6	19.0	2248.9	19.8	2200.2	22.1
**LPC 20:0**					411.1	17.4		
**LPC 20:1**					591.5	32.6	459.1	31.6
**LPC 20:3**	633.6	18.2	1846.8	44.4	2571.2	29.7	2257.4	31.3
**LPC 20:4**	648.8	18.3	1673.7	36.3	4901.6	64.4	1801.3	43.2
**LPC 20:5**			1828.2	17.9	1005.2	60.4	682.9	35.0
**LPC 22:4**					499.7	27.9		
**LPC 22:6**	382.7	6.4	3927.7	28.0	848.9	77.3	1845.7	42.4
**LPE 16:0**	517.1	24.0	1414.9	45.8	1599.5	60.1	1178.0	45.5
**LPE 18:0**	753.7	30.0	1689.5	48.4	3468.4	56.1	3077.4	46.6
**LPE 18:1**			594.1	25.7	2889.3	42.7	1195.2	51.5
**LPE 18:2**			385.8	40.8	674.5	45.9	423.3	29.4
**LPE 18:3**					403.9	32.5		
**LPE 20:0**			430.7	40.8	371.0	14.6		
**LPE 20:1**					381.7	44.7		
**LPE 20:3**			467.7	33.7	700.2	32.2	581.4	35.8
**LPE 20:4**			460.2	31.8	1311.7	54.5	495.2	42.6
**LPE 22:6**			1591.0	28.2	756.5	52.4	1271.0	50.8
**LPG 16:0**	3549.7	12.6	2536.5	9.9	2912.9	9.2	2305.9	13.0
**LPG 16:0+NH4**	647.7	27.0	489.8	11.7	515.9	12.8	421.7	12.7
**LPG 18:3**	1180.7	9.4	951.2	8.6	1107.1	9.9	979.5	11.7
**LPI 24:0**	2852.7	5.0	2415.4	5.1	2092.1	9.9	1724.7	19.4
**PC 26:1**	16315.2	9.8	13811.4	31.9	12645.9	50.6	14450.4	25.5
**PC 30:0**			461.0	37.2	346.1	44.7	521.6	42.4
**PC 30:1**							346.0	31.6
**PC 32:0**	3279.0	37.3	4537.7	51.7	4368.3	45.2	7851.1	81.3
**PC 32:1**	13926.8	56.4	13184.8	36.4	3377.4	17.0	3892.8	33.1
**PC 32:2**	1862.0	42.9	1839.2	22.9	1237.2	21.2	2033.3	51.9
**PC 34:1**	54391.3	16.3	56713.6	12.1	43037.2	11.1	45408.5	18.0
**PC 34:2**	66018.3	8.5	70735.8	5.5	67013.0	8.2	64016.2	13.8

**PC 34:3**	7288.3	36.7	7448.1	17.6	8741.8	27.3	6270.6	27.6
**PC 34:4**	2898.5	38.5	2603.7	20.6	1547.7	16.3	997.0	27.4
**PC 34:5**	471.3	57.7	426.8	11.0				
**PC 36:0**							419.2	31.6
**PC 36:1**	7898.7	57.3	5728.5	33.8	6969.1	32.3	8198.2	55.4
**PC 36:2**	37699.6	23.2	41875.7	11.1	54707.0	15.6	50875.0	19.2
**PC 36:3**	19578.0	66.2	14857.9	29.3	54117.9	13.5	39005.0	30.6
**PC 36:4**	43576.2	23.9	23436.0	8.4	53708.5	12.4	17932.3	27.6
**PC 36:5**	7642.7	14.6	43538.4	8.5	7168.2	15.2	22586.3	35.6
**PC 36:6**	2126.8	25.3	2842.6	9.8	2101.8	25.3	2344.6	19.8
**PC 38:1**	201.4	173.2	409.9	40.8	478.9	14.6	429.0	23.4
**PC 38:2**	1440.1	48.8	1459.4	18.7	1446.7	40.8	1148.9	36.5
**PC 38:3**	3229.9	58.3	1989.9	31.3	14573.3	32.5	6066.9	60.1
**PC 38:4**	21699.2	29.5	8511.0	20.8	41940.9	17.5	11106.9	37.4
**PC 38:5**	9438.8	37.0	19060.3	22.9	39693.1	25.1	17142.9	40.2
**PC 38:6**	38269.6	25.3	65190.7	2.9	27251.5	19.8	62401.7	9.4
**PC 38:7**	3425.6	25.5	6822.0	7.4	3822.6	10.7	6462.8	22.2
**PC 38:8**	946.8	41.8	4890.6	7.0	1010.3	29.0	2741.1	27.8
**PC 40:10**	943.0	31.0	998.1	11.1	407.4	20.0	1139.4	21.5
**PC 40:3**					387.1	17.5		
**PC 40:4**	911.8	39.0	462.7	40.8	1264.1	39.6	498.5	30.5
**PC 40:5**	991.0	39.7	1282.6	29.7	5430.8	25.9	679.3	50.9
**PC 40:6**	9695.0	28.4	26071.3	11.9	12134.6	27.0	37494.7	13.0
**PC 40:7**	4508.9	29.3	5835.4	12.6	9151.3	18.5	23058.8	24.4
**PC 40:8**	1690.6	32.5	5008.2	15.9	4029.3	15.1	5480.5	24.7
**PC 40:9**	3929.8	11.6	5740.9	2.0	3020.8	13.8	5896.9	9.2
**PC 42:10**	548.7	35.2	754.2	4.9	878.5	18.6	2158.4	18.2
**PC 42:11**	0.0		714.9	24.9	456.2	26.3	1175.9	14.0
**PC 42:6**	926.9	30.7	714.0	19.4	472.3	24.2	636.3	35.2
**PC 42:9**	1065.0	24.9	2384.5	12.2	1241.7	19.8	3103.9	12.4
**PC 44:12**			593.3	12.3			1088.3	34.0
**PE 32:0**							358.2	18.5
**PE 32:1**							545.3	30.1
**PE 34:1**	703.4	35.9	536.5	34.6	674.5	19.1	627.7	31.2
**PE 34:2**	3454.1	26.9	2842.2	19.1	4871.1	20.5	2385.3	49.6
**PE 34:3**	529.8	57.7			568.0	28.1	369.2	31.6
**PE 36:1**	1101.2	39.2	1165.1	20.9	667.5	33.8	775.3	53.9
**PE 36:2**	2599.8	24.5	2819.2	14.4	4954.2	18.6	4010.2	37.8
**PE 36:3**	1247.7	34.4	953.4	14.2	4418.9	16.5	2113.6	42.8
**PE 36:4**	7920.4	46.7	2312.8	21.3	8504.4	21.1	1669.7	39.7
**PE 36:5**	1198.3	37.4	3338.4	7.9	1036.0	17.7	1954.2	41.2
**PE 36:6**							404.3	8.4
**PE 38:1**	556.7	19.8	654.4	16.2			389.1	15.8
**PE 38:2**	1034.7	30.8	1203.7	14.2	1007.0	14.5	853.9	28.4
**PE 38:3**			380.2	7.6			449.1	12.3

**PE 38:4**	18324.3	34.7	7423.8	11.8	27983.5	14.8	7699.9	49.6
**PE 38:5**	4012.2	42.1	7062.7	11.6	17455.8	24.8	7449.5	34.5
**PE 38:6**	14973.5	34.5	24081.4	6.8	12345.9	21.9	30262.5	21.7
**PE 38:7**	1422.7	34.9	1210.9	12.1	1438.5	16.5	1621.7	26.8
**PE 38:8**			540.1	3.3			439.1	13.3
**PE 40:2**	476.5	20.3	550.2	20.9	383.8	18.6	370.7	9.4
**PE 40:4**	909.2	28.4	616.3	11.9	1085.6	22.1	425.6	18.5
**PE 40:5**	712.6	26.6	1187.5	10.1	3205.8	23.2	389.1	1.7
**PE 40:6**	5700.7	32.2	13523.3	8.7	7542.8	21.4	24932.0	22.7
**PE 40:7**	4302.5	33.7	4292.8	10.9	7472.2	16.4	13630.8	40.1
**PE 40:8**	923.8	31.2	1298.7	7.7	2306.2	13.1	2151.5	31.6
**PE 40:9**	955.7	8.9	1364.6	5.7	825.2	7.8	1750.4	8.5
**PE 42:11**			587.9	9.7			991.3	22.6
**PE 42:6**	577.4	18.5	1120.3	21.8	462.4	15.5	786.7	13.0
**PE 42:9**	682.5	21.2	1279.4	4.9	749.4	13.6	1757.6	14.6
**PE 44:12**							771.3	20.6
**PE 44:6**			75.6	244.9				
**PG 32:0**	771.2	5.6	682.4	6.7	593.9	16.7	666.5	13.6
**PG 34:0**	1412.7	11.8	1300.1	10.0	1227.6	12.9	1275.7	14.3
**PG 34:1**							514.4	48.6
**PG 36:0**					395.0	44.7		
**PG 36:3**	418.4	57.7	369.0	40.8	467.5	7.8	358.2	37.7
**PG 38:6**					441.3	20.6		
**PG 40:6**			410.4	40.8				
**PI 38:4**	404.0	57.7			641.9	22.1	438.5	18.6
**PI 38:4+NH4**					438.0	16.9	435.3	31.6
**PI 38:5**							370.8	39.5
**PI 38:5+NH4**							387.8	52.3
**PI 38:7**	762.9	24.1	519.6	10.7	623.1	22.1	151.8	129.7
**PI 40:6**							418.0	50.0
**PI 40:7**	474.2	11.0	363.9	40.8	928.3	9.2	580.8	10.3
**PI 40:8**			368.5	6.1	598.0	18.8	439.7	54.7
**PI 40:9**			477.2	5.9			514.3	16.2
**PI 42:6**	467.8	29.5	385.5	40.8	476.9	13.9	433.4	37.7

Average intensities of phospholipids with relative standard deviations (SD) of all TAG detected in cyclohexane extract of mouse liver using positive mass spectrometry ionzation mode.

(blank boxes mean not detected)

C – control; CP – control and n-3-PUFA; LPC – lysophosphatidylcholine; LPE – lysophosphatidylethanolamine; M – MCD diet; MCD – high fat methionine choline-deficient diet; MP – MCD diet + n-3-PUFA; PE – phosphatidylethanolamine; PC – phosphatidylcholine; PE – phosphatidylethanolamine; PG – phosphatidylglycerol; PI – phosphatidylinositol.

Table S2. The effect of n-3-PUFA on liver phospholipids - negative mass spectrometry ionzation mode.

	C		CP		M		MP	
	average intensity	SD (%)	average intensity	SD (%)	average intensity	SD (%)	average intensity	SD (%)
**LPE 16:0**	186.9	12.2	560.0	55.2	730.2	50.3	578.8	52.4
**LPE 16:1**					87.8	17.0		
**LPE 18:0**	241.3	16.4	702.4	59.3	1534.0	62.8	1474.9	56.2
**LPE 18:1**	106.4	51.0	232.8	39.6	1543.1	38.9	642.8	56.4
**LPE 18:2**			88.0	35.7	206.8	46.5	102.6	50.4
**LPE 20:0**			66.9	40.8	85.9	21.4	75.5	21.9
**LPE 20:1**					122.0	43.8	84.3	22.1
**LPE 20:2**	111.5	7.6	125.0	5.8	142.4	7.9	125.4	6.8
**LPE 20:4**	76.0	32.6	94.1	38.6	377.2	55.5	98.1	48.2
**LPE 22:6**	86.8	25.6	585.4	32.7	255.7	56.1	550.3	60.8
**LPI 16:0**	65.2	57.7	109.0	19.6			75.8	15.2
**LPI 18:0**	253.4	60.7	515.6	30.5	596.9	48.5	930.3	43.1
**LPI 18:1**					171.0	19.4	120.6	40.6
**LPI 20:3**							87.1	31.6
**LPI 20:4**			66.6	29.1	234.6	39.3	82.0	25.8
**LPI 24:0**			48.7	40.8			56.0	4.6
**LPI 24:1**	50.8	57.7	53.3	7.1	62.9	8.6	62.9	8.2
**PE 32:1**	49.6	57.7						
**PE 34:1**	106.9	37.9	70.6	26.4	101.1	15.9	90.3	32.6
**PE 34:2**	383.0	24.6	317.5	17.6	627.6	21.6	330.3	52.1
**PE 34:3**	56.9	57.7			73.8	28.0	0.0	
**PE 36:1**	100.1	32.0	101.7	22.0	118.0	18.6	125.7	40.6
**PE 36:2**	332.8	22.0	343.7	14.2	751.3	18.7	576.8	40.7
**PE 36:3**	140.0	36.5	105.9	11.9	551.4	15.4	284.1	46.8
**PE 36:4**	806.1	41.0	229.8	16.7	1048.6	24.0	181.3	50.9
**PE 36:5**	99.1	45.0	401.9	10.8	74.9	20.9	237.2	45.3
**PE 38:2**	71.4	23.8	60.9	15.8	127.3	31.6	98.4	21.7
**PE 38:3**					89.3	26.7	97.7	45.4
**PE 38:4**	2071.9	36.8	806.9	18.1	3652.1	27.6	919.9	45.7
**PE 38:5**	380.3	42.7	716.7	15.9	2003.7	20.2	822.2	40.3
**PE 38:6**	1400.3	30.3	2543.7	5.8	1317.2	15.6	3319.5	32.7
**PE 38:7**	70.2	39.2	78.4	12.0	84.3	14.7	155.3	33.1
**PE 40:4**	83.3	30.6			131.9	12.7		
**PE 40:5**	86.1	57.7	92.8	14.4	538.4	27.4		
**PE 40:6**	712.5	35.6	1517.8	10.5	1230.9	18.9	3743.6	34.9

**PE 40:7**	222.1	33.4	319.6	10.3	611.1	9.6	1454.0	35.9
**PE 40:8**	53.7	57.7	52.8	40.8	88.5	19.4	140.3	44.4
**PE 42:1**					97.8	44.7	75.5	31.6
**PE 42:6**	49.8	57.7	69.7	20.0	73.1	24.4	89.8	26.0
**PE 42:7**							90.4	41.2
**PG 34:1**	95.9	33.8	82.9	11.9	122.2	12.2	101.0	40.3
**PG 34:2**							76.8	31.6
**PG 36:2**					110.7	11.6	70.7	15.7
**PG 36:3**					148.1	10.8	113.6	31.6
**PG 36:4**					94.8	11.8	75.2	36.8
**PG 38:4**					64.2	44.7		
**PG 38:5**					60.5	44.7		
**PG 40:6**					108.7	24.0		
**PG 40:7**					84.9	9.2	73.3	29.4
**PG 40:8**							81.2	31.6
**PG 44:10**					55.1	44.7		
**PG 44:12**			113.5	40.7	0.0		208.4	49.9
**PI 34:2**	131.7	43.9	196.1	16.9	96.2	44.4	126.3	51.2
**PI 36:2**	106.2	39.2	126.5	13.9	179.9	41.0	231.6	36.5
**PI 36:3**			55.7	14.4	108.4	32.4	110.4	45.3
**PI 36:4**	898.5	73.0	546.0	17.7	554.0	39.9	267.3	53.9
**PI 36:5**			135.0	16.2			99.1	41.5
**PI 38:3**							355.9	31.6
**PI 38:4**	2792.3	46.6	2119.3	19.8	8429.6	32.8	4353.8	36.5
**PI 38:5**	121.0	52.1	391.4	25.2	946.2	22.9	943.4	37.9
**PI 38:6**	61.8	57.7	138.3	15.9			202.8	38.3
**PI 40:3**	130.8	25.7	119.2	23.7	153.5	26.3	153.8	22.0
**PI 40:4**	59.2	21.5	65.7	40.8	94.3	30.1	89.8	50.1
**PI 40:5**			72.3	23.2	85.7	21.3	166.6	34.6
**PI 40:6**	66.9	57.7	215.7	36.6			715.3	47.9
**PI 40:7**							101.4	42.5
**PI 44:12**					18.6	223.6		

Average intensities of phospholipids with relative standard deviations (SD) of all glycerophospholipids detected in cyclohexane extract of mouse liver using negative mass spectrometry ionzation mode.

C – control; CP – control and n-3-PUFA; LPC – lysophosphatidylcholine; LPE – lysophosphatidylethanolamine; M – MCD diet; MCD – high fat methionine choline-deficient diet; MP – MCD diet + n-3-PUFA; PE – phosphatidylethanolamine; PC –

phosphatidylcholine; PE – phosphatidylethanolamine; PG – phosphatidylglycerol; PI – phosphatidylinositol.

(blank boxes mean not detected)
